# Supplementary material for: Exogenous Cannabinoids Impair Effort-Related Decision-Making via Affecting Neural Synchronization between the Anterior Cingulate Cortex and Nucleus Accumbens
Source: Brain Sci. 2023 Feb 27;13(3):413. doi: 10.3390/brainsci13030413 (PMC10046840; doi:10.3390/brainsci13030413)
Supplement: Supplementary file 1 [file brainsci-13-00413-s001.zip › brainsci-2158957-supplementary.pdf]

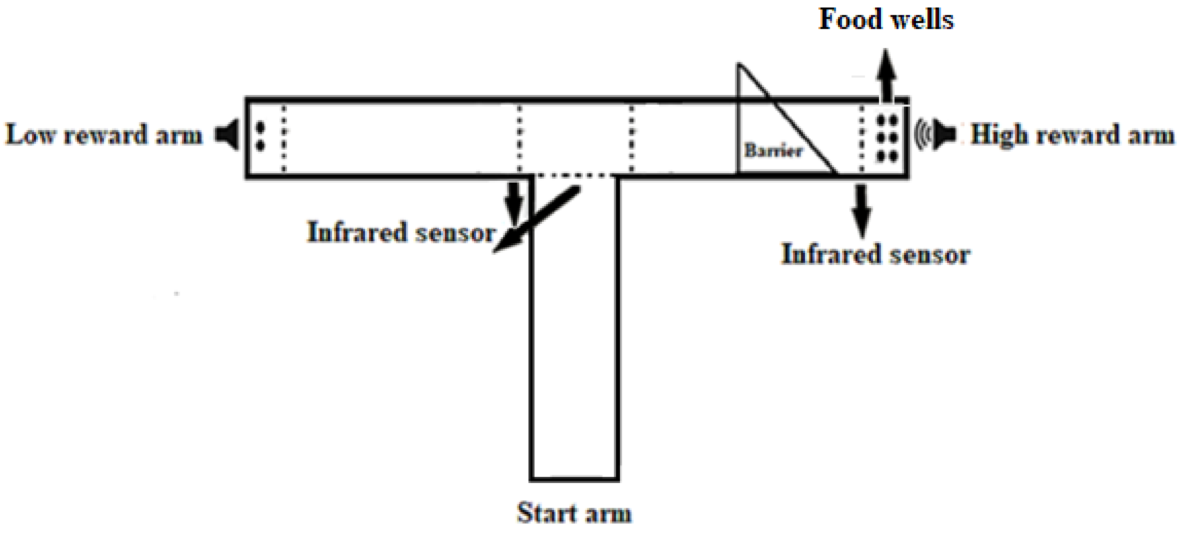

Supplementary Figure S1A. Schematic illustration of the apparatus. Effort-based T-maze decision-making task. The apparatus has three arms including start, high reward and low reward arms. Rats could choose to climb a barrier (30 cm) in order to obtain a higher reward (6 piece of reward), and/or choose to receive a small reward (2 piece of reward) without physical effort. There are five infrared sensors. One of them is placed at the end of start arm, two of them are at the beginning of the goal arms, and two others are at the beginning of food wells. Once the animals broke the infrared sensor at the end of start arm, an audio signal played from a speaker at the end of the high reward arm to determine the high reward arm for the animals. When the animals entered goal arm, they broke the infrared sensor at the beginning of goal arm. Besides, when the animals reached food wells, they broke the infrared sensor at the beginning of food wells.

Behavioral training has three phases

Habituation phase: the rats were located at the start arm, and for 20 minutes, they were free to explore all over the maze.

Discrimination phase: the rats learned how to discriminate between low-rewarded and high-rewarded target arms.

For highlighting high remuneration arm, once the animals broke the infrared sensor at the end of start arm, an audio signal played from a speaker at the end of the high remuneration arm to determine the high remuneration arm for the animals.

Barrier phase: barrier was put in the middle of the high remuneration arm. Training animals in decision-making task until animals prefer high reward choice over 80% for three following days.

Supplementary Figure S1B. Schematic paradigm of Behavioral training.

Surgical procedure and implanting electrode.

↓

Rats were retrained to maze until preferring HRC over 80% (to sure they prefer HRC as before surgery).

↓

On test day, the animals received, DMSO, and/or agonist, and/or antagonist+agonist, and after 5 minutes, they performed 10 trials decision-making task. Local field potential recording was recorded for all trials.

↓

When the animals entered goal arm, they broke the infrared sensor at the beginning of goal arm. LFP signals 500 ms before and 500 ms after broking IR were analyzed.

When the animals broke IR at the beginning of food wells, LFP signals 500 ms before and 500 ms after that were analyzed.

Supplementary Figure S1C. Schematic paradigm of experimental design.

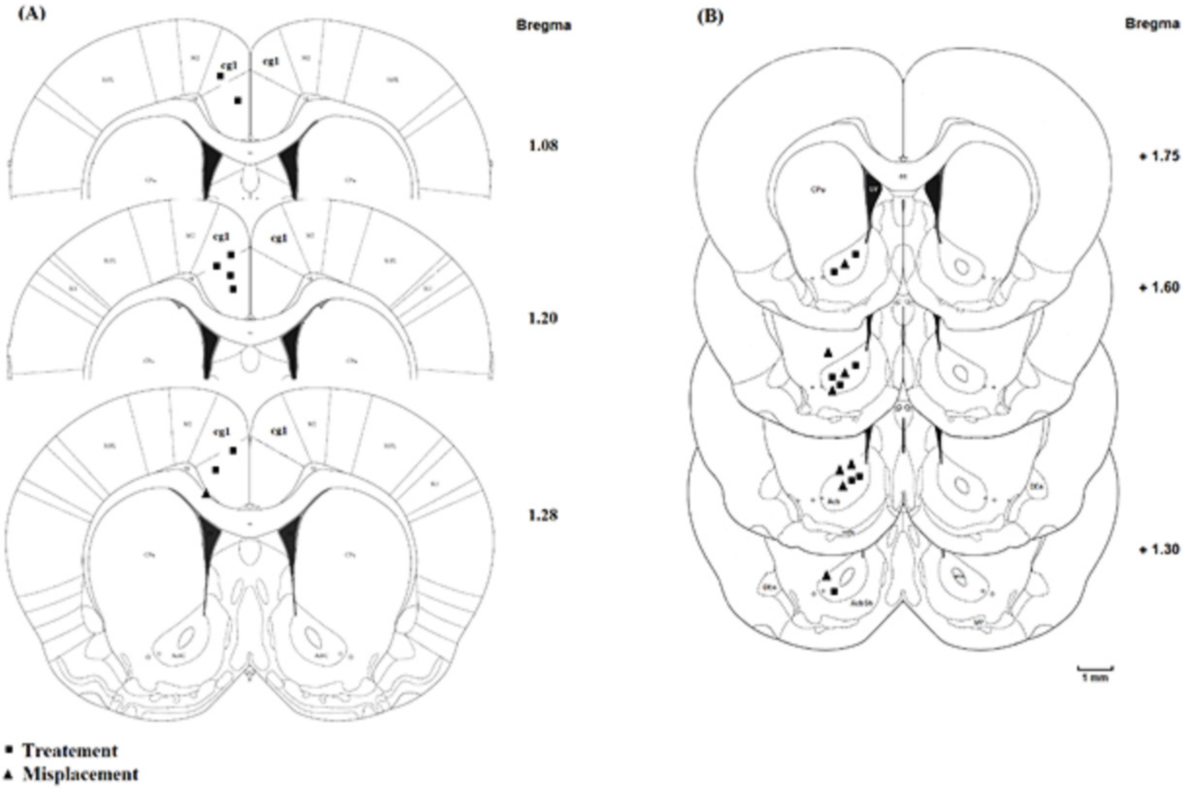

Supplementary Figure S2: Anatomical reconstruction of electrode tips in the ACC **(A)** and NAc (core) **(B)**. Corrected electrode tips, Misplacement.

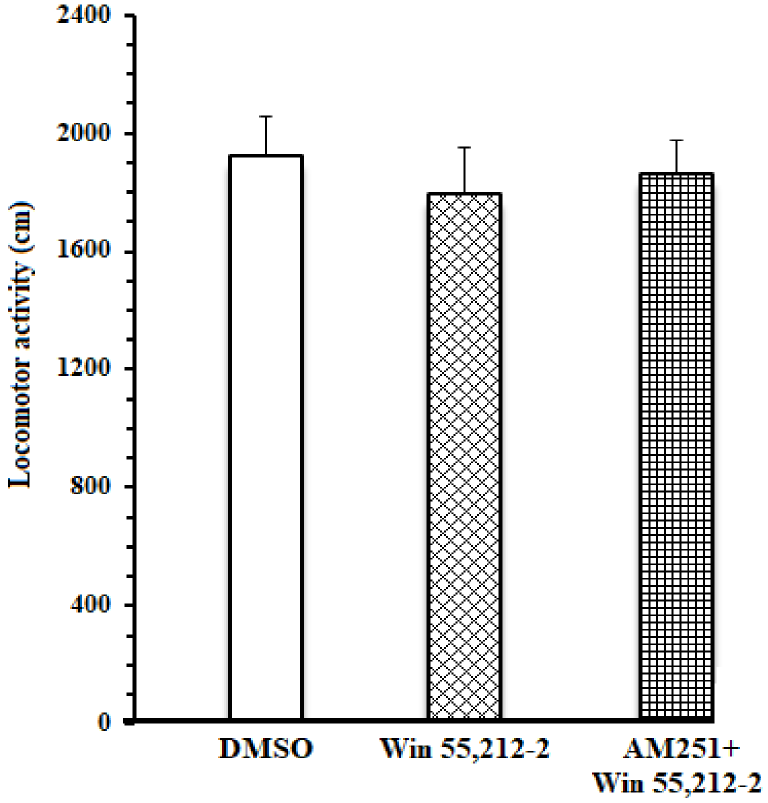

Supplementary Figure S3A. Administration of Win 55,212-2 and AM251 into the NAc did not have any significant effect on locomotor activity. Data show as mean ± SEM for 8 rats.

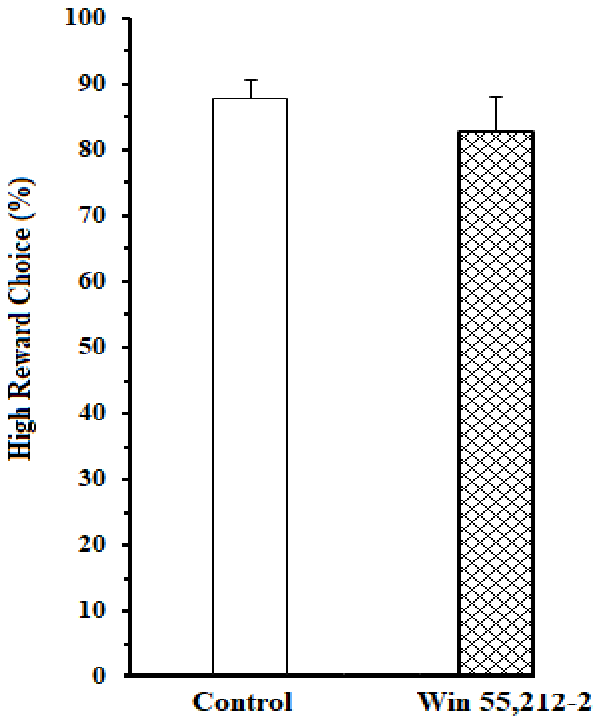

Supplementary Figure S3B. The performance on a control task in which the same group of rats had passed the barrier to receive reward in both goal arms. Data show as mean ± SEM for 7 rats.

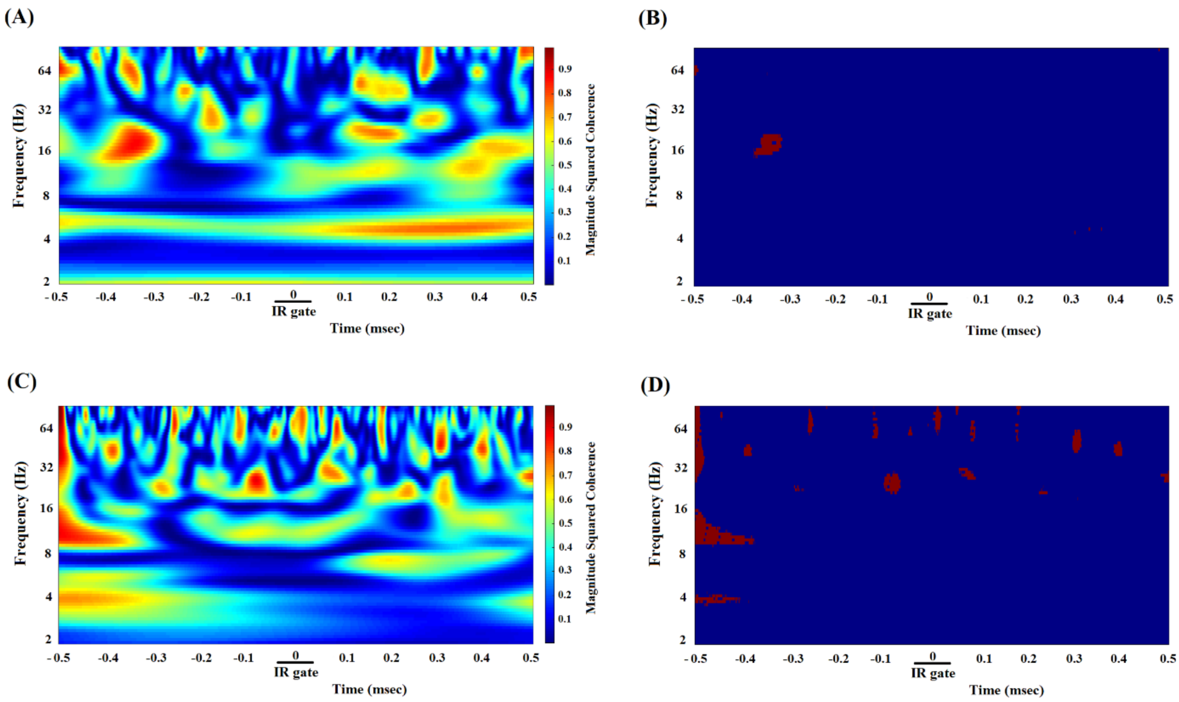

Supplementary Figure S4. Comparison of coherence of group averages between low remuneration/low endeavor trials in the control group during make a decision **(A)**, and while reaching remuneration **(C)**. Time-frequency representation of group averages coherence between the ACC and NAc. The cone of influence (COI) where edge effects should be considered is blurred. It should be noted that the wavelet scales are converted to approximate frequencies on the graphs. Comparison of areas in which the coherence is significantly different compared to a large number of surrogate data between low remuneration/low endeavor trials in the control group during make a decision **(B)**, and while reaching a remuneration **(D)**. The red regions in the figures show the areas with a significant difference ($P < 0.05$). Surrogate data were simulated using bootstrapping ($n = 1000$).
